# Immediate implant placement with and without platelet-rich fibrin and decalcified freeze-dried bone graft

**DOI:** 10.6026/973206300214430

**Published:** 2025-12-15

**Authors:** Akriti Agrawal, Abdullah M Alwaisi, Awaiz Ahmed, Azza A Abushama, Noor Yasir, Shahnaaz Sultana, Md Kafeel Ahmed

**Affiliations:** 1 Department of Periodontology and Implantology, Consultant, P.N. Singhdeo Dental Clinic, Sambalpur, Odisha, India; 2 Department of Periodontology and Implantology, Gulf Medical University, Ajman, Aljurf 1, UAE; 3 Department of Pediatric Dentistry, Consultant, Dr. Awaiz's Dental Planet, Bidar, Karnataka, India; 4 Department of Preventive Dental Sciences (Periodontology) College of Dentistry, Dar Al Uloom University, Riyadh, Saudi Arabia; 5 Department of Periodontology and Implantology, Al Badar Rural Dental College and Hospital, Kalaburagi, Karnataka, India; 6 Department of Periodontology and Implantology, MNR Dental College and Hospital, Sangareddy, Hyderabad, Telangana, India

**Keywords:** Immediate implant placement, platelet-rich fibrin, bone grafting, DFDBA, implant stability, dental esthetics

## Abstract

There is a high risk of vertical and horizontal bone resorption in the peri-implant gap following immediate implant placement after non-molar tooth extraction, which compromises implant stability, hard/soft tissue preservation, and esthetic outcomes. The present study was a randomized controlled clinical trial included 40 patients requiring a single non-molar tooth extraction and immediate implant placement. Patients were randomly allocated into two equal groups (n=20). In the Test group, the peri-implant gap was filled with a composite of PRF and DFDBA. In the Control group, no grafting material was placed. The primary outcome was vertical bone level (VBL) change at 6 months, measured on standardized radiographs. Secondary outcomes included horizontal bone thickness changes measured on CBCT, implant stability quotient (ISQ) at baseline and 3 months and Pink Esthetic Score (PES) at 6 months. Independent t-tests were used for statistical analysis (α=0.05). The adjunctive use of a composite PRF and DFDBA graft in immediate implant placement sockets significantly improves the preservation of peri-implant hard and soft tissues, enhances secondary implant stability and leads to superior esthetic outcomes compared to no grafting.

## Background:

Immediate implant placement into fresh extraction sockets has become a widely accepted treatment modality in implant dentistry. This approach offers several advantages, including a reduction in overall treatment time, fewer surgical interventions and the potential for preserving the existing alveolar bone architecture [1]. By placing the implant at the time of tooth extraction, the psychological and functional burden on the patient is minimized. Despite its benefits, the protocol is not without challenges, the most significant of which is the management of the inevitable dimensional changes that occur in the alveolar ridge following tooth removal [2]. Upon extraction, the bundle bone that forms the socket wall undergoes rapid resorption, primarily affecting the thin buccal plate. This process can lead to vertical bone loss and horizontal ridge collapse, which in turn compromises the long-term stability and esthetic outcome of the implant restoration, often resulting in soft tissue recession and exposure of the implant collar [3]. A key factor influencing this outcome is the presence of a horizontal discrepancy, or "jumping distance," between the implant surface and the inner walls of the extraction socket. The debate continues as to whether this gap should be filled with a bone grafting material to support the tissues and promote regeneration [4, 5]. Various biomaterials have been proposed to fill this peri-implant gap, with allografts such as decalcified freeze-dried bone allograft (DFDBA) being a popular choice. DFDBA serves as an osteoconductive scaffold, preventing soft tissue collapse into the defect and providing a framework for new bone formation. Furthermore, the decalcification process potentially exposes bone morphogenetic proteins (BMPs), giving it osteoinductive properties that can actively stimulate bone regeneration [6, 7]. In recent years, the focus has shifted towards incorporating biological modifiers to enhance the healing process. Platelet-rich fibrin (PRF), a second-generation platelet concentrate, is an autologous biomaterial prepared through a simple centrifugation protocol [8]. The resulting fibrin matrix is rich in platelets, leukocytes and a multitude of growth factors, including platelet-derived growth factor (PDGF), transforming growth factor-beta (TGF-β) and vascular endothelial growth factor (VEGF). These factors are released slowly over 7-14 days, promoting angiogenesis, cell proliferation and differentiation, thereby accelerating both soft and hard tissue healing [9, 10]. Combining PRF with a bone graft material like DFDBA is hypothesized to create a "composite graft" that possesses both the structural scaffolding of the graft and the regenerative stimulus of the growth factors [11]. The clinical evidence comparing a composite PRF/DFDBA graft against a non-grafted control group in immediate implant sockets is limited and its impact on hard tissue preservation, implant stability and final esthetic outcomes has not been fully elucidated. Therefore, it is of interest to evaluate and compare the clinical, radiographic and esthetic outcomes of immediate implant placement in fresh extraction sockets with and without the use of a composite graft of platelet-rich fibrin and decalcified freeze-dried bone allograft.

## Materials and Methods:

## Study design and sample selection:

This study was a single-center, parallel-group, randomized controlled clinical trial conducted between June 2021 and December 2022. A power analysis was conducted based on data from a previous study. To detect a clinically significant difference of 0.5 mm in vertical bone loss with a standard deviation of 0.4 mm, a power of 80% and an alpha of 0.05, a sample size of 17 patients per group was required. To account for potential dropouts, 20 patients were recruited for each group, resulting in a total sample of 40 patients. Inclusion criteria were: (1) age between 20 and 65 years; (2) requiring a single tooth extraction in the maxillary or mandibular premolar region; (3) presence of intact buccal and lingual/palatal socket walls; (4) sufficient bone (>4 mm) beyond the root apex for primary implant stability; (5) adequate oral hygiene. Exclusion criteria included: (1) uncontrolled systemic diseases; (2) pregnancy or lactation; (3) history of head and neck radiation; (4) use of bisphosphonates; (5) active periodontal infection or acute periapical lesion at the site; (6) heavy smoking (>10 cigarettes/day); (7) parafunctional habits like bruxism.

## Surgical and restorative protocol:

All surgical procedures were performed by a single calibrated surgeon. After local anesthesia, the designated tooth was extracted as atraumatically as possible without flap elevation. The socket was thoroughly debrided. The osteotomy was prepared according to the manufacturer's protocol and a tapered, tissue-level implant (NobelActive, Nobel Biocare, Switzerland; 4.3 mm diameter, 10 or 11.5 mm length) was placed. Primary stability was confirmed by achieving an insertion torque of at least 35 Ncm. Participants were then randomized using a computer-generated allocation sequence concealed in sealed opaque envelopes.

[1] Control Group (n=20): The implant was placed, a cover screw was connected and the peri-implant gap was left to heal by blood clot formation. The gingival margins were approximated with a single 5-0 polypropylene suture.

[2] Test Group (n=20): Immediately before surgery, 10 mL of venous blood was drawn from the patient and centrifuged at 2700 rpm for 12 minutes (Intra-Spin, Intra-Lock International) to prepare PRF clots according to Choukroun's protocol. The PRF clots were compressed to form membranes. A portion of the membrane was minced and mixed with DFDBA particles (Puros Cortical Particulate Allograft, Zimmer Biomet; particle size 0.25-1.0 mm) to form a sticky composite graft. This graft was gently packed into the peri-implant gap. A full PRF membrane was then placed over the graft and tucked under the gingival margins. A cover screw was placed and the site was sutured.

All patients received post-operative instructions, including amoxicillin 500 mg three times daily for 7 days, ibuprofen 600 mg as needed for pain and a 0.12% chlorhexidine gluconate rinse twice daily for two weeks.

## Outcome assessments:

All measurements were performed by a blinded and calibrated examiner.

[1] Primary Outcome: Vertical Bone Level (VBL) Change: Standardized periapical radiographs were taken using a long-cone paralleling technique at baseline (day of surgery) and at the 6-month follow-up. VBL was measured from the implant shoulder to the first visible bone-to-implant contact on both mesial and distal aspects using digital software (ImageJ, NIH, USA). The change in VBL was calculated.

[2] Secondary Outcomes:

1. Horizontal Bone Thickness Change: Cone-beam computed tomography (CBCT) scans (Carestream CS 9300) were taken at baseline and 6 months using a low-dose protocol. The change in horizontal thickness of the buccal bone plate was measured at a level 1 mm apical to the implant platform.

2. Implant Stability Quotient (ISQ): ISQ values were measured using a resonance frequency analysis device (Osstell ISQ, W & H) at the time of implant placement (baseline) and at 3 months post-operatively.

3. Pink Esthetic Score (PES): At the 6-month follow-up, standardized clinical photographs were taken after provisional restoration. PES was evaluated by two independent, calibrated prosthodontists based on seven variables (mesial papilla, distal papilla, soft-tissue level, soft-tissue contour, alveolar process deficiency, soft-tissue color and texture). The maximum possible score is 14.

## Statistical analysis:

Data were analyzed using SPSS Statistics v.26 (IBM Corp., Armonk, NY). The normality of data distribution was checked using the Shapiro-Wilk test. Independent samples t-tests were used to compare the mean values between the two groups for all parameters. Paired t-tests were used for intragroup comparisons between time points. The level of significance was set at p < 0.05.

## Results:

All 40 enrolled patients (22 female, 18 male; mean age 45.8 ± 9.2 years) completed the 6-month follow-up period. No implants were lost, resulting in a 100% survival rate in both groups. There were no significant differences in baseline demographics or site characteristics between the groups. At the 6-month follow-up, the Test group demonstrated significantly less vertical bone loss compared to the Control group on both mesial and distal aspects. The mean mesial VBL change was 0.45 ± 0.18 mm in the Test group versus 1.15 ± 0.25 mm in the Control group (p < 0.001). Similarly, the mean distal VBL change was 0.48 ± 0.21 mm in the Test group compared to 1.21 ± 0.28 mm in the Control group (p < 0.001). The results are summarized in Table 1 (see PDF). The secondary outcomes also showed statistically significant differences favoring the Test group. The change in horizontal buccal bone thickness measured on CBCT was significantly less in the Test group (-0.5 ± 0.3 mm) compared to the Control group (-1.4 ± 0.4 mm) (p < 0.001). Baseline ISQ values were comparable between the groups. However, at 3 months, the mean ISQ was significantly higher in the Test group (75.8 ± 3.1) than in the Control group (71.2 ± 4.5) (p = 0.012). The esthetic outcome, as measured by PES at 6 months, was significantly better in the Test group, with a mean score of 12.1 ± 1.5, compared to 9.8 ± 2.1 in the Control group (p = 0.008). These findings are presented in Table 2 (see PDF) and Table 3 (see PDF).

## Discussion:

The findings of this randomized controlled trial demonstrate that grafting the peri-implant gap with a composite of PRF and DFDBA during immediate implant placement offers significant advantages over no grafting. The primary hypothesis was confirmed, as the Test group showed substantially less vertical and horizontal bone loss, improved secondary implant stability and superior esthetic outcomes at the 6-month follow-up. The preservation of peri-implant bone levels is paramount for the long-term success of dental implants. In our study, the Control group, where the gap was left to heal with a blood clot alone, experienced over 1 mm of vertical bone loss. This is consistent with previous literature reporting significant ridge remodeling following tooth extraction [2, 3]. The Test group, however, experienced less than 0.5 mm of bone loss, a value well within the criteria for implant success. This superior outcome can be attributed to the synergistic action of the graft components. The DFDBA particles provided a physical scaffold that prevented the collapse of the socket walls and maintained space for new bone formation [7], while the PRF enriched this scaffold with a sustained release of growth factors that actively stimulated osteogenesis and angiogenesis [12]. This is supported by studies that show PRF, when combined with bone grafts, can accelerate bone regeneration and maturation [11, 13]. A critical finding was the significant preservation of the horizontal buccal bone thickness in the Test group. The buccal plate is particularly susceptible to resorption and its maintenance is crucial for supporting the overlying soft tissue and achieving an esthetic result [14]. The -0.5 mm resorption in the Test group compared to -1.4 mm in the Control group highlights the efficacy of the composite graft in mitigating this collapse. This hard tissue preservation translated directly into better soft tissue outcomes, as evidenced by the significantly higher Pink Esthetic Scores in the Test group. The improved scores for papilla fill, soft tissue contour and alveolar process profile in the grafted group underscore the intimate relationship between underlying bone and overlying gingival architecture. The biostimulatory effect of PRF on gingival fibroblasts may have also contributed to enhanced soft tissue healing and maturation [15, 16]. The implant stability measurements provide further insight into the healing dynamics. While both groups achieved excellent primary stability, the Test group showed significantly higher ISQ values at 3 months. This suggests an accelerated rate of osseointegration. The growth factors within PRF, particularly PDGF and TGF-β, are potent stimulators of osteoblast activity, likely leading to faster bone apposition onto the implant surface and a more rapid increase in secondary stability [10, 17]. This could be clinically advantageous, potentially allowing for earlier loading protocols in select cases. This study has several strengths, including its randomized controlled design, blinded outcome assessment and evaluation of multiple clinically relevant parameters. However, some limitations should be acknowledged. The follow-up period was limited to 6 months; long-term data are necessary to confirm if these benefits are maintained over time. The study was also limited to non-molar sites with intact sockets, so the results may not be generalizable to sites with bone defects or to molar extraction sockets. Finally, while CBCT provides accurate 3D assessment, its use involves radiation exposure and is not routinely indicated for post-operative follow-up.

## Conclusion:

Grafting the peri-implant gap in immediate implant placement with a composite of platelet-rich fibrin and decalcified freeze-dried bone allograft significantly reduces both vertical and horizontal peri-implant bone loss compared to no grafting. The use of this composite graft leads to a significant increase in secondary implant stability at 3 months post-surgery. The preservation of hard tissue architecture in the grafted group results in significantly better soft tissue esthetic outcomes, as measured by the Pink Esthetic Score.
